# Supplementary material for: Early binding of anti-amyloid antibodies to CAA drives complement activation, inflammation and ARIA in mice
Source: Mol Neurodegener. 2026 Jun 26;21:57. doi: 10.1186/s13024-026-00965-x (PMC13579800; doi:10.1186/s13024-026-00965-x)
Supplement: Supplementary file 2 [file 13024_2026_965_MOESM1_ESM.docx]

**Supplementary Fig.1: Acute anti-Aβ immunization indicates early preferential binding of 3D6 to vascular amyloid**. **a,** Acute passive immunotherapy in 25-month-old *APP^NL-G-F^* KI mice with 20mg/kg 3D6 mAb on Day 1 and 4, followed by euthanasia on Day 7. Controls received IgG2a isotype control mAb. Immunostaining for S97 (general Aβ) and anti Ms-IgG2a (to detect 3D6 mAb) shows that 3D6 preferentially binds to amyloid (arrows) in the cortical and cerebellar meninges and large vessels near the ventricles. 3D6 can be seen in the choroid plexus as well (arrowhead). **b,** Brain sections stained with Amylo-Glo, Collagen-IV and anti-Mouse IgG2a. Cerebellar CAA-associated mAb binding is indicated by arrowheads, plaque labelling by asterisks**,** and non-CAA-associated vascular mAb near ventricles (right image panel) is indicated by a colored arrowhead**.** Unbound mAb in the parenchyma outside CAA vessels is indicated by an arrow**. c,** qPCR analysis for complement genes *C4* & *C3* from the bulk cerebellum tissue, Mann-Whitney test, *p < 0.05, data are expressed as mean ± SEM. Scale bar in a =200 µm, b = 50 µm.

**Supplementary Fig.2:** **Passive anti-Aβ immunization for 7 weeks was associated with differentially expressed genes in the FACS-sorted CD31^+^ endothelial cells in the brain. a,** Schematic for anti-Aβ immunotherapy in the 20-month-old *APP/PS1dE9;hAPOE4* mice with endothelial cell isolation, RNA extraction followed by Smart-seq2 (n=3, 2 Males and 1 Female mouse). **b,** Volcano plot showing differentially expressed genes. **c,** Pheatmap plot showing selected significantly expressed genes related to cholesterol efflux (*Abca1*, *Ch25h*) and blood homeostasis (*Vwf)* and complement genes *(Cfh)* and others related to lipid stress and inflammation, denoted with arrowheads. **d,** Bar plots representing the biological pathways that are down and up-regulated.

**Supplementary Fig.3: qPCR analysis of relevant genes associated with complement pathway and BBB integrity, following 7 weeks of passive anti-Aβ immunization** in the 20-month-old *APP/PS1dE9;hAPOE4* mice. RNA was extracted, and qPCR was performed on the cerebellum tissue. Statistical analysis was conducted using a Parametric unpaired t-test *p<0.05. Data are expressed as mean ± SEM.

**Supplementary Fig.4:** **Anti-Aβ immunotherapy and associated microhemorrhages. a,** MRI imaging was performed in 20-month-old *APP/PS1dE9;hApoE4* mice following 13 weeks of 500 µg/wk (15 mg/kg body weight) 3D6 mAb passive immunization and Prussian blue hemosiderin staining on the brain tissue sections after euthanasia. **b**, Representative Prussian blue stained brain sections from two 3D6 treated mice and their corresponding MRI sagittal planes (approximate) showing hemosiderin deposits and matched hypointense spots as indicated by circles or arrows.

**Supplementary Fig.5: Chronic anti-Aβ passive immunization with 3D6 mAb caused increased recruitment of CD206+ border-associated macrophages (BAM) along C3 deposited areas and mAb-associated vWF deposition in aged 20-mo-old *APP/PS1dE9:hAPOE4* mice following 13 weeks of passive immunization with 500 µg/wk mAb**. **a,** Schematic of passive immunization **b,** Representative Immunofluorescence staining in the cerebellum area for C3, GFAP, CD206, and Amylo-Glo (AG). ImageJ quantification of % CD206+ area in the **c,** Isocortex (cortex & hippocampus) and **d,** Cerebellum area. C3+CD206+ colocalized area in the **e,** Isocortex and **f,** Cerebellum area in IgG2a and 3D6 treated mice (n=4-5/group). Data represented as mean ± SEM. Parametric test with unpaired t-test ***p=0.0005 and ****p<0.0001. **g**, Immunostaining of brain sections with von Willebrand factor (vWF), a glycoprotein produced by endothelial cells or platelets, anti-mouse IgG2a, and amyloid staining with Amylo-Glo. Scale bar: b=200 µm, g= 25 µm.
